# *N*-Formylated Peptide Induces Increased Expression of Both Formyl Peptide Receptor 2 (Fpr2) and Toll-Like Receptor 9 (TLR9) in Schwannoma Cells—An In Vitro Model for Early Inflammatory Profiling of Schwann Cells

**DOI:** 10.3390/cells9122661

**Published:** 2020-12-11

**Authors:** Andrea Korimová, Petr Dubový

**Affiliations:** Cellular and Molecular Neurobiology Research Group, Department of Anatomy, Faculty of Medicine, Masaryk University, CZ-62500 Brno, Czech Republic; 442669@mail.muni.cz

**Keywords:** Wallerian degeneration, mitochondria, disintegration, damage-associated molecular patterns, cytokines, chemokines, receptors

## Abstract

Following nerve injury, disintegrated axonal mitochondria distal to the injury site release mitochondrial formylated peptides and DNA that can induce activation and inflammatory profiling of Schwann cells via formyl peptide receptor 2 (Fpr2) and toll-like receptor 9 (TLR9), respectively. We studied RT4 schwannoma cells to investigate the regulation of Fpr2 and TLR9 after stimulation with fMLF as a prototypical formylated peptide. RT4 cells were treated with fMLF at various concentrations and times with and without pretreatment with inhibitors (chloroquine for activated TLR9, PBP10 for Fpr2). Western blots of Fpr2, TLR9, p-p38, p-NFκB, and IL-6 were compared in relation to inflammatory profiling of RT4 cells and chemokine receptors (CCR2, CXCR4) as potential co-receptors of Fpr2. fMLF stimulation upregulated Fpr2 in RT4 cells at low concentrations (10 nM and 100 nM) but higher concentrations were required (10 µM and 50 µM) when the cells were pretreated with an activated TLR9 inhibitor. Moreover, the higher concentrations of fMLF could modulate TLR9 and inflammatory markers. Upregulation of Fpr2 triggered by 10 nM and 100 nM fMLF coincided with higher levels of chemokine receptors (CCR2, CXCR4) and PKCβ. Treating RT4 cells with fMLF, as an in vitro model of Schwann cells, uncovered Schwann cells’ complex responses to molecular patterns of release from injured axonal mitochondria.

## 1. Introduction

Wallerian degeneration (WD) is a cascade of cellular and molecular events distal to a nerve injury. It is considered an innate immune reaction or a sterile inflammation. Schwann cells in contact with degenerated axons are activated and reprogrammed towards axon promotion [1]. This activation of a post-injury phenotype in Schwann cells is linked to their inflammatory profiling to generate the conditions necessary for axon regeneration [2,3,4,5,6]. Distal segments of the injured axons contain disintegrated mitochondria [7,8,9,10] releasing both mitochondrial proteins and DNA (mtDNA), collectively characterized as mitochondrial damage-associated molecular patterns (mtDAMPs) [11,12].

Inflammatory profiling of Schwann cells, exhibited as upregulation of cytokines/chemokines, [6,13] can be triggered by Toll-like receptors (TLRs), which are activated by cell fragments and other molecules produced by WD distal to a peripheral nerve injury [14,15]. Similar to bacterial DNA, mtDNA has unmethylated CpG DNA motifs that are ligands for TLR9 and can mediate inflammatory reaction through NFκB [12]. In contrast to membrane-bound TLRs, TLR9 is located in the endoplasmic reticulum and is translocated to endosomal membranes following stimulation by CpG DNA [16].

Disintegrated mitochondria of injured axons, like other trauma-damaged cells, release short *N*-formylated peptides [12,17] that serve as chemo-attractants for immune cells and can trigger inflammatory profiling of tissue cells following traumatic injury [17,18,19,20]. *N*-formylated peptides are considered the canonical ligands for *N*-formyl peptide receptors (FPRs). The human family of FPRs has three members: FPR1, FPR2/ALX (formerly termed FPR-like 1 or FPRL1), and FPR3 (formerly FPRL2) [21].

*N*-formyl-methionyl-leucyl-phenylalanine (fMLF) is a prototypical *N*-formylated peptide and, as such, has been used in numerous studies of its role in activation of innate immune reactions including those in the nervous system [22,23,24]. Nevertheless, little is known about its function in activating Schwann cells. Fpr2, the rodent orthologue of human FPR2/ALX, shows structural and functional similarities [25,26] including low-efficiency binding towards the prototypical fMLF [27,28].

The rat RT4-D6P2T schwannoma cell line is derived from the *N*-ethyl-*N*-nitrosourea induced rat peripheral neurotumor RT4. The RT4 cell line has been widely and effectively employed as a model for studying the molecular processes and signaling machinery in Schwann cells [29,30,31,32], mainly due to easy in vitro cultivation, in contrast to primary Schwann cell culture.

The aim of present study was to investigate early regulation of the protein levels and signaling functionalities of Fpr2 and TLR9 in the RT4 schwannoma cell model after treatment with fMLF as a canonical Fpr2 ligand and its role as a potential molecular stimulus for inflammatory profiling of Schwann cells. fMLF stimulation was carried out at various concentrations and times. Moreover, we also tested whether fMLF at a particular concentration can modulate levels of potential Fpr2 co-receptors such as the chemokine receptors CCR2 and CXCR4.

## 2. Materials and Methods

### 2.1. Cell Culture and fMLF Treatment

The rat RT4-D6P2T schwannoma cell line (RT4; ATCC-LGC, Sesto San Giovanni, Italy) was maintained in Dulbecco’s Modified Eagle’s Medium/Nutrient F-12 Ham (DMEM/F12) supplemented with 10% fetal bovine serum (FBS), 2 mM l-glutamine, and antibiotics (100 U/mL penicillin, 100 μg/mL streptomycin) at 37 °C and 5% CO_2_ atmosphere. Prior to all experiments, the culture near confluence was seeded in cultured medium onto 6 cm plastic tissue culture dishes at a density such that cultures did not reach confluence before the end of the test to minimize the risk of over confluence at the time of analysis. After attachment, the cells were washed twice with PBS and starved in serum-free medium following the treatments. All media and supplements were from Sigma Aldrich (Sigma-Aldrich, s.r.o., Prague, Czech Rep).

To investigate inflammatory profiling of RT4 cells induced by mitochondrial formyl peptides, the cells were stimulated with fMLF (Sigma-Aldrich) at final concentrations of 100 nM, 10 μM, and 50 μM for 1 and 6 h. fMLF was dissolved in dimethyl sulfoxide (DMSO), therefore, equal concentrations of DMSO were added to the medium in control cell cultures (100 nM, 0.002%; 10 µM, 0.2%; 50 µM, 1%). The fMLF–mediated synergic activation of Fpr2 and TLR9 was tested by pre-incubation of RT4 cells with 1 μM chloroquine (CQ, InvivoGen, Toulouse, France) for 30 min to inhibit TLR9 prior to fMLF treatment (Table 1). Pretreatment of RT4 cells with CQ effectively blocks the active endosomal form of TLR9 [33].

We saw that fMLF triggered increased levels of Fpr2 only at 100 nM for 6 h, therefore, RT4 cells in the second experiment were stimulated with fMLF at a different range of concentrations (10 nM, 100 nM, 1 μM), and pretreatment of RT4 cells with 1 μM PBP10 (Tocris, Bristol, UK) for 20 min was used to test specificity of Fpr2 stimulation (Table 1). The control cells of this set of experiments were treated with corresponding concentrations of DMSO (10 nM, 0.0002%; 100 nM, 0.002%; 1 µM, 0.02%).

### 2.2. Western Blot Analysis

After cultivation in the presence or absence of stimulants or inhibitors for different time periods, cells were washed twice with ice-cold phosphate-buffered saline, scraped off, and then collected by gentle centrifugation. The samples were lysed in a buffer containing 80 mM HEPES, pH 7.5; 2.5 M urea, 1 mM EDTA, 0.5% Triton X-100, and 20 mM β-mercaptoethanol supplemented with cocktails of protease and phosphatase inhibitors (Roche, Mannheim, Germany). Protein concentrations were determined using the Bradford assay (Bio-Rad s.r.o., Prague, Czech Rep.). Equal amounts of proteins from cell lysates (50 µg/lane) were separated by 10% sodium dodecyl sulfate-polyacrylamide gel electrophoresis and then transferred to a nitrocellulose membrane (Bio-Rad s.r.o., Prague, Czech Rep.). After adding 5% non-fat dry milk or 5% bovine serum albumin for blocking in TRIS-buffered saline-0.2% Tween 20 buffer (TBS-T) for 1 h at room temperature with agitation, the membranes were incubated with primary antibodies (Table 2) at 4 °C overnight. The membranes were washed with TBS-T, incubated for 1 h at room temperature with peroxidase-conjugated goat anti-rabbit or anti-mouse secondary antibodies (1:1000, Millipore, Tamecula, CA, USA), and visualized using a chemiluminescent substrate and ECL (Bio-Rad s.r.o., Prague, Czech Rep.) on a chemiluminometer reader (Syngene Pxi Trigon-plus). The bands were measured using image densitometry software (Gene Tools from Syngene, Cambridge, UK) and normalized to β-actin (1:1000, Cell Signaling, Leiden, The Netherlands) for semiquantitative evaluation.

### 2.3. Statistical Analysis

All experiments were repeated at least three times. The data of Western blot analysis were expressed as means ± SEM and compared using the non-parametric Mann–Whitney U-test in GraphPad Prism 7 (GraphPad Software, San Diego, CA, USA). We considered *p* values less than 0.05 significant. Because DMSO was used as a solvent and the vehicle for fMLF, we compared data of Western blot analysis of RT4 cells after fMLF treatment to those of cells cultivated in medium supplemented only with DMSO as controls.

## 3. Results

### 3.1. Fpr2 and TLR9 Protein Levels in RT4 Cells Following fMLF Stimulation

We analyzed Fpr2 protein levels in whole-cell lysate prepared from RT4 schwannoma cells by Western blots using a commercially available rabbit polyclonal antibody (NLS1878, Novus Biologicals, Centennial, CO, USA) detecting a protein band at 38 kDa corresponding to the molecular weight of Fpr2.

**Figure 1:**
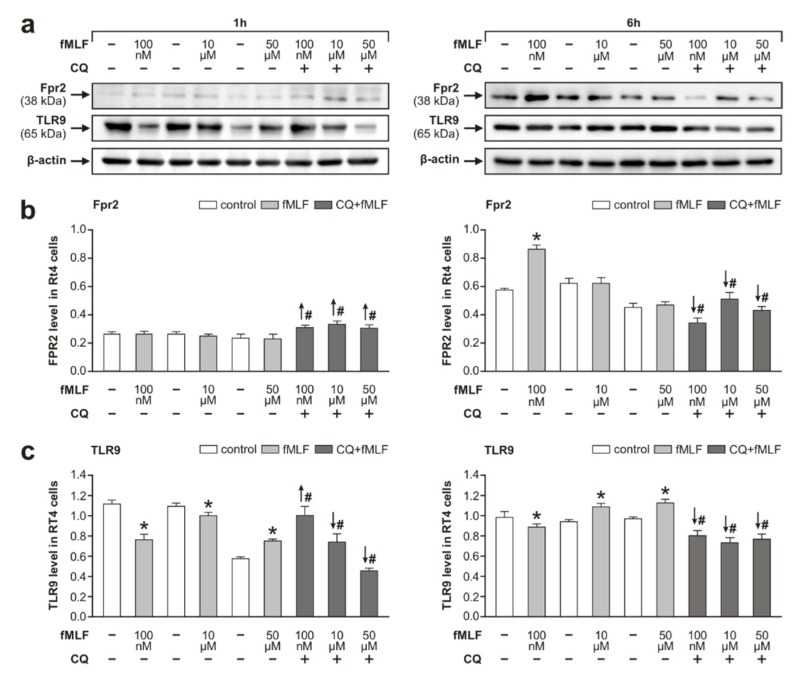
Effect of *N*-formyl-methionyl-leucyl-phenylalanine (fMLF) stimulation on the levels of Fpr2 and TLR9 in RT4 cells. Protein levels of Fpr2 and TLR9 in RT4-D6P2T cells treated with different concentrations of fMLF (100 nM, 10 µM, and 50 µM) for 1 and 6 h and pretreatment with CQ (1 µM) were analyzed using Western blots. Panel (**a**) shows representative Western blots of TLR9, Fpr2, and β-actin. Results of densitometric measurements of bands from triplicate Western blot analysis normalized to the housekeeping protein β-actin are depicted in the graphs in panels (**b**,**c**). Results are shown as means ± SEM, * *p* < 0.05 compared to control, # *p* < 0.05 compared to stimulation with the relevant fMLF concentration without 1 µM CQ, the up and down arrows indicate increased and decreased levels, respectively.

No significant changes of the band densities at 38 kDa were detected after fMLF stimulation at the concentrations of 100 nM, 10 μM, or 50 μM for 1 h compared with that of the control cells treated with DMSO alone. After fMLF treatment for 6 h, we observed a significantly increased level of Fpr2 only at 100 nM, while the other fMLF concentrations showed no effect on Fpr2 protein levels (Figure 1a,b).

Although fMLF is not considered a ligand of TLR9, we tested the effect of fMLF on TLR9 as the other receptor type that reacts to mtDAMPs. We detected the 65 kDa band corresponding to the cleaved active form of TLR9 responsible for its interaction with MyD88 and subsequent signaling [34]. Interestingly, we saw a significant decrease in TLR9 levels after fMLF stimulation at 100 nM and 10 μM, but 50 μM fMLF acting for 1 h significantly increased TLR9 levels. In contrast, the fMLF stimulation for 6 h resulted in increased levels of the cleaved TLR9 form at 10 μM and 50 μM, whereas its level was decreased only at 100 nM fMLF when compared to that of controls. This decreased level of TLR9 upon treatment with 100 nM fMLF coincided with increased levels of Fpr2 (Figure 1a,c).

We also monitored changes in the levels of Fpr2 and TLR9 following fMLF stimulation in parallel experiments where RT4 cells were pretreated with 1 μM CQ, an inhibitor of the active form of TLR9 [33]. Pretreatment with 1 μM CQ before fMLF stimulation for 1 h significantly increased levels of Fpr2, but the same pretreatment before 10 μM or 50 μM fMLF stimulation for 1 h significantly decreased the levels of the cleaved form of TLR9 compared to that of cells without the pretreatment. In contrast, CQ pretreatment of RT4 cells followed by a longer fMLF stimulation (for 6 h) resulted in a significant decrease of both Fpr2 and TLR9 protein levels compared to cells without the pretreatment (Figure 1a–c).

### 3.2. Fpr2 and TLR9 Molecular Signaling in RT4 Cells Following fMLF Stimulation

**Figure 2:**
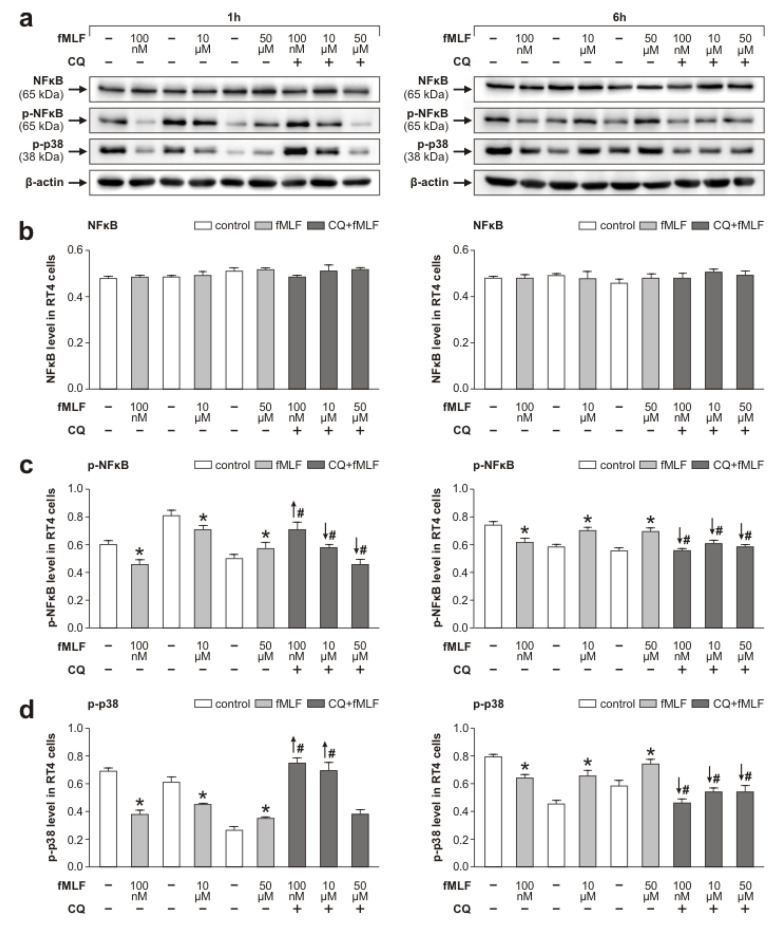
Western blot analysis of activated NFκB and p38 in RT4 cells after their stimulation with *N*-formyl-methionyl-leucyl-phenylalanine (fMLF). Changes in NFκB, p-NFκB, and p-p38 protein levels in RT4-D6P2T cells following fMLF stimulation at 100 nM, 10 µM, and 50 µM for 1 and 6 h and pretreatment with CQ (1 µM) are shown. Representative Western blots of NFκB, p-NFκB, and p-p38 as well as housekeeping protein β-actin of three independent experiments are seen in panel (**a**). The graphs illustrate values expressed as means ± SEM of densitometric measurements for NFκB (**b**), p-NFκB (**c**) and p-p38 (**d**) bands after 1 h and 6 h fMLF stimulation and pretreatment with CQ (1 µM) from triplicate Western blot analyses of RT4 cells. * *p* < 0.05 compared to control, # *p* < 0.05 compared to stimulation with the relevant fMLF concentration without 1 µM CQ, the up and down arrows indicate increased and decreased levels, respectively.

Fpr2 and TLR9 signaling pathways in glial cells involve activation of p38 MAPK and NFκB, respectively [27,35]. To investigate the molecular signaling through which fMLF can influence inflammatory profiling of RT4 cells, we first investigated NFκB activation. No significant changes of NFκB levels were detected following fMLF stimulation at any concentration either for 1 h or 6 h. We also found no changes in NFκB when RT4 cells were pretreated with CQ compared to its levels in cells without the pretreatment (Figure 2a,b).

We found changes in the phosphorylated p65 subunit of NFκB (p-NFκB) in response to fMLF stimulation at 100 nM, 10 μM, and 50 μM concentrations for 1 h and 6 h (Figure 2a,c). The dynamics of the changes in p-NFκB was similar to the changes in TLR9 following fMLF treatment for 1 and 6 h with and without CQ pretreatment (Figure 1a,c). That is, levels of p-NFκB were decreased by fMLF treatment at 100 nM and 10 μM for 1 h, but were increased at 50 μM fMLF acting for 1 h. fMLF treatment for 6 h resulted in decreased levels only at 100 nM, while 10 μM and 50 μM fMLF increased p-NFκB levels compared to that of controls. Pretreatment with CQ reduced p-NFκB in RT4 cells following 10 μM and 50 μM fMLF stimulation while at 100 nM fMLF the level of p-NFκB was significantly higher when compared to that of cells without CQ pretreatment. Similar to TLR9, CQ pretreatment followed by fMLF stimulation for 6 h significantly reduced p-NFκB levels (Figure 2a,c).

We also analyzed the activation of p38 in fMLF-stimulated RT4 cells in the same set of experiments where we monitored Fpr2 and the cleaved form of TLR9. The pattern of phosphorylated p38 (p-p38) levels compared to controls was similar to changes in TLR9 and p-NFκB levels observed in RT4 cells following fMLF stimulation for 1 h and 6 h. In addition, CQ pretreatment followed by 100 nM and 10 μM fMLF stimulation for 1 h increased p-p38 levels compared to levels without CQ pretreatment. However, CQ pretreatment followed by fMLF stimulation for 6 h significantly reduced p-p38 in RT4 cells, showing the same CQ effect as on p-NFκB levels (Figure 2a,d).

### 3.3. Inflammatory Profiling of RT4 Cells Following fMLF Stimulation

**Figure 3:**
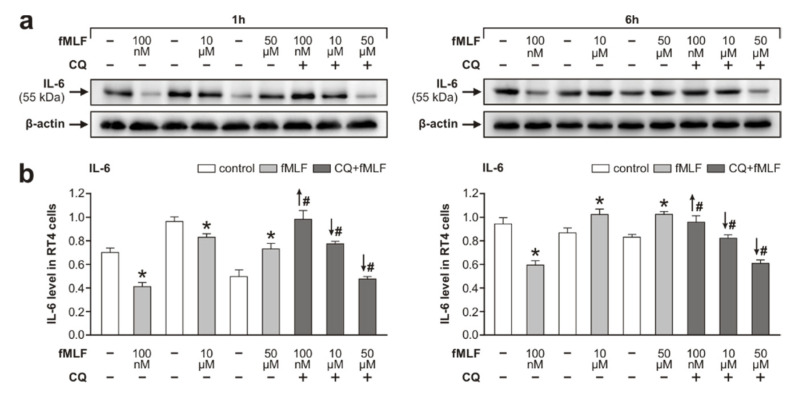
Western blot analysis of IL-6 protein levels in RT4-D6P2T cells after stimulation of *N*-formyl-methionyl-leucyl-phenylalanine (fMLF) at 100 nM, 10 µM, and 50 µM for 1 and 6 h and pretreatment with CQ (1 µM). Panel (**a**) shows representative Western blots of IL-6 and β-actin as the housekeeping protein. The graphs (**b**) depict densitometric measurements of triplicate Western blots expressed as means ± SEM. * *p* < 0.05 compared to control, # *p* < 0.05 compared to stimulation with the relevant fMLF concentration without 1 µM CQ, the up and down arrows indicate increased and decreased levels, respectively.

We used Western blot analysis of IL-6 protein levels to investigate fMLF-induced inflammatory profiling of RT4 cells. fMLF-induced changes in IL-6 protein levels in RT4 cells were similar to that of fMLF-induced changes in p-NFκB and p-p38. Levels of IL-6 were significantly lowered by 100 nM and 10 μM fMLF treatment for 1 h, but they were increased upon treatment with 50 μM fMLF for 1 h compared to controls. In contrast, 100 nM fMLF treatment for 6 h resulted in significantly lower levels of IL-6 compared to that of control, but a higher concentration of fMLF (10 μM and 50 μM) triggered significantly increased levels of IL-6 compared to that of controls. Pretreatment with CQ demonstrated a similar effect on IL-6 levels following fMLF treatment for 1 h and 6 h (Figure 3a,b).

### 3.4. Effect of fMLF on Levels of Chemokine Receptor CCR2 and CXCR4

We saw that fMLF triggered increases in the level of Fpr2 only at a concentration of 100 nM for 6 h; in a second round of experiments, we used RT4 cells treated with a different range of fMLF concentrations (10 nM, 100 nM, and 1 μM) for 6 h. In addition, the specificity of the fMLF effect on the level of Fpr2 was tested by pretreating RT4 cells with PBP10, a selective agonist of Fpr2 [36]. This set of experiments was also used to study the regulation of CCR2 and CXCR4 as putative co-receptors for formylated peptides.

**Figure 4:**
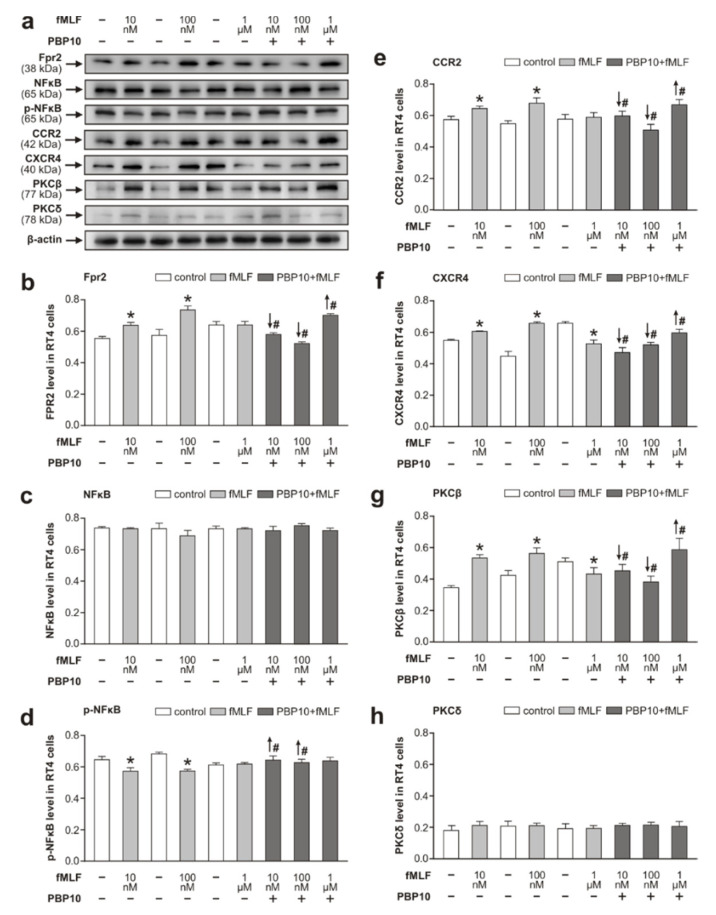
Parallel dynamics of Fpr2 and chemokine receptors CCR2 and CXCR4 and their signaling. Western blot analysis of Fpr2, NFκB, p-NFκB, CCR2, CXCR4, PKCβ, and PKCδ in RT4-D6P2T cells stimulated with *N*-formyl-methionyl-leucyl-phenylalanine (fMLF) at 10 nM, 100 nM, and 1 μM and after pretreatment with PBP10 (1 µM). Panel (**a**) contains representative Western blots of the analyzed proteins and β-actin as a housekeeping protein. The graphs depict densitometric measurements of triplicate Western blots expressed as means ±SEM for Fpr2 (**b**), NFκB (**c**), p-NFκB (**d**), CCR2 (**e**), CXCR4 (**f**), PKCβ (**g**), and PKCδ (**h**). * *p* < 0.05 compared to control, # *p* < 0.05 compared to stimulation with the relevant fMLF concentration without 1 µM PBP10, the up and down arrows indicate increased and decreased levels, respectively.

We confirmed that RT4 cells treated with 100 nM fMLF for 6 h significantly increased Fpr2 protein levels compared to that of the control. Further, stimulation with 10 nM fMLF for 6 h also increased Fpr2 protein levels, but no effect was found when the concentration was increased to 1 μM. The increase in Fpr2 seen at 10 nM and 100 nM fMLF was significantly reduced by pretreatment of RT4 cells with PBP10, while a higher concentration (1 μM), fMLF overcame the inhibitory effect of PBP10 and induced a significantly increased level of Fpr2 compared to that of cells without the pretreatment (Figure 4a,b).

As in the previous set of experiments with cells treated with higher fMLF concentrations for 1 and 6 h (Figure 2), no significant changes in NFκB levels were found in this set of experiments (Figure 4a,c). However, levels of activated p-NFκB dropped in RT4 cells following treatment with 10 nM and 100 nM fMLF for 6 h compared to that of controls. PBP10 pretreatment followed by fMLF stimulation at 10 nM and 100 nM resulted in significantly increased levels of p-NFκB compared to that of cells without the pretreatment (Figure 4a,d).

The alteration in CCR2 and CXCR4 protein levels in RT4 cells following fMLF treatment as well as in cells pretreated with PBP10 (Figure 4a,e,f) was very similar to the results of the Western blots monitoring Fpr2 levels (Figure 4a,b,e,f). 

As the binding of ligands to Fpr2 and chemokine receptors triggers several intracellular signaling cascades including PKC isoforms [37,38], we investigated whether PKCβ or PKCδ participate in the intracellular signaling of fMLF stimulation in RT4 cells. Western blot analysis revealed that PKCβ, not PKCδ, was the PKC isoform associated with fMLF-mediated activation of Fpr2, CCR2, and CXCR4. Interestingly, alteration in PKCβ levels in cells that were given a PBP10 pretreatment and fMLF stimulation was similar to results of Fpr2, CCR2, and CXCR4 Western blot analysis (Figure 4a,b,e–g). However, we found very low levels of PKCδ and the change in its level following fMLF stimulation with or without PBP10 pretreatment was not significant (Figure 4a,h).

## 4. Discussion

Wallerian degeneration events distal to the nerve injury, usually considered a sterile inflammation, include inflammatory profiling of Schwann cells that is characterized by upregulation of cytokines [6]. The cytokines in Schwann cells may modulate neurotrophins to promote axon regeneration [39,40]. In distal segments of injured axons, mitochondria are disintegrated and release mtDAMPs including, among others, mtDNA and formylated peptides that trigger intracellular signaling via TLR9 and Fpr2, respectively [27,28,41].

### 4.1. Effects of fMLF Stimulation on Upregulation of Fpr2 and Modulation of TLR9 in RT4 Cells

*N*-formyl peptides are cleavage products of bacterial and mitochondrial proteins that serve as chemo-attractants for immune cells and can trigger inflammatory profiling of tissue cells after traumatic injury [17,19,20,42]. *N*-formyl peptide receptors are transmembrane proteins belonging to the G-protein-coupled receptor family. Rodent Fpr2 is activated by an array of ligands including formyl peptides and their prototype fMLF [27,28]. Recently, it was demonstrated that rat Schwann cells treated with Annexin A1 display increased level of Fpr2 mRNA and activation of Fpr2 [43]. We hypothesized that inflammatory profiling of Schwann cells similar to immune cells [44] can be modulated by formylated peptide activation of Fpr2 present in the Schwann cells distal to nerve injury [45].

The RT4-D6P2T cell line represents the immortalized schwannoma cells with the expression of key genes that characterize primary Schwann cells [46]. Another advantage of RT4-D6P2T cells is that they provide a homogeneous population free of the cellular senescence present very early in primary Schwann cell cultures [47]. RT4 schwannoma cells were used as an in vitro model of Schwann cells to investigate the course of cytoplasmic events following stimulation of fMLF and CpG [45]—ligands for Fpr2 and TLR9, respectively [36,41]. Our objective was to extend our knowledge about the reaction of RT4 cells (used as an in vitro model of Schwann cells) exposed to various concentrations of fMLF for varying lengths of time.

Because rodent Fpr2 is defined as a low-affinity fMLF receptor based on its activation only by micromolecular concentrations of fMLF [23,48], we at first tested stimulation of RT4 cells with 100 nM, 10 µM, and 50 µM fMLF concentrations. These experiments revealed significantly increased levels of Fpr2 in RT4 cells stimulated with 100 nM fMLF for 6 h. Although we used a specific anti-Fpr2 antibody, we cannot rule out a contribution of Fpr1 in the reaction of RT4 schwannoma cells to the fMLF effect.

Living cells react to mtDAMPs released from damaged cells via various types of pattern-recognition receptors including formyl peptide receptors and TLRs [49,50,51]. It is generally believed that formyl peptide receptors bind various lipids and formylated peptides [23,48], and that sequences of mtDNA are ligands for TLR9 [41]; cooperation between these different types of receptors have been experimentally demonstrated. For example, it was evidenced in vitro that CpG ODN, a TLR9 ligand, may also upregulate microglial Fpr2 levels [52]. Furthermore, synergy between TLR3 or TLR7 and Fpr2 was demonstrated in microglial cells [53]. However, to our knowledge, the results we present are the first demonstration that fMLF as a prototype of formylated peptides can modulate TLR9 in glial cells in a concentration-dependent manner. Indeed, fMLF treatment of RT4 cells also evoked a concentration-dependent modulation of the cleaved form of activated TLR9. Our results indicate interaction of Fpr2 and TLR9 in RT4 cells following fMLF stimulation. We conclude this from the following:

- the upregulation of Fpr2 stimulated with 100 nM fMLF coincided with a simultaneous decrease in the activated form of TLR9,
- the level of Fpr2 increased in RT4 cells stimulated with fMLF for 1 h following TLR9 inhibition, and
- in contrast, fMLF stimulation for 6 h following inhibition of the active TLR9 form significantly reduced the level of Fpr2.

Atypical modulation of TLR9 by fMLF stimulation was associated with inflammatory profiling of RT4 cells. This was demonstrated by similar changes of TLR9 and activated p38, NFκB, and the level of IL-6 in RT4 cells following fMLF stimulation for both 1 and 6 h (Figure 1, Figure 2 and Figure 3). Phosphorylation of p38 and NFκB is an integral component of signal transduction after activation of both Fpr2 and TLR9 [27,54,55]. Moreover, it is also well-known that activated p38 and NFκB participate in endogenous signaling pathways leading to the synthesis of cytokines [56,57].

fMLF stimulation via Fpr2 and TLR9 in RT4 cells shows a contradictory effect on IL-6 levels in RT4 cells. fMLF stimulation at 100 nM concentration produced a decrease of IL-6 when compared to that of control RT4 cells, but when the stimulation was applied after the inhibition of TLR9, it resulted in the elevation of IL-6. On the other hand, the enhanced levels of IL-6 seen following fMLF stimulation at higher concentrations (10 and 50 µM) for 6 h were significantly reduced when preceded by a CQ pretreatment (Figure 3). The results may indicate that 100 nM fMLF activation of Fpr2 has an inhibitory effect on IL-6 and inflammatory profiling of RT4 cells, but fMLF at a higher concentration (10 and 50 µM) increased IL-6 and inflammatory profiling of these cells acting via TLR9. In addition, the anti-inflammatory effect of fMLF via Fpr2 activation was also found in our second set of experiments, where the fMLF concentrations were 10 nM and 100 nM. fMLF stimulation at these concentrations reduced the activation of NFκB, but it was abolished by a preceding Fpr2 inhibition (Figure 4a,b,d). These results are in line with an anti-inflammatory role for activated Fpr2 as seen in immune cells with additional regulatory functions [48,58].

### 4.2. Cooperation of Fpr2 and Chemokine Receptors CCR2 and CXCR4 in RT4 Cells Following fMLF Stimulation

When our first experiments with fMLF stimulation at 100 nM, 10 µM, and 50 µM for 1 and 6 h revealed significantly increased levels of Fpr2 in RT4 cells only at 100 nM fMLF for 6 h, we performed another set of experiments with RT4 cells stimulated with fMLF at 10 nM, 100 nM, and 1 µM. This set of experiments confirmed that nanomolar concentrations of fMLF can induce significantly increased levels of Fpr2 in RT4 cells. The efficiency of fMLF at nanomolar concentrations to induce increased levels of Fpr2 was unequivocally proven by the effect of PBP10, an antagonist of Fpr2. These results are in contradiction to the experiments with immune cells that characterized rodent Fpr2 as a low-affinity receptor activated by micromolar concentrations of fMLF [27,59]. 

The second set of experiments with stimulated RT4 cells was also used to investigate the role of chemokine receptors CCR2 and CXCR4 as co-receptors of fMLF-stimulated Fpr2. Both Fpr2 and chemokine receptors belong to the G-protein-coupled receptor superfamily [60,61] whose signal transduction pathways include protein kinase C (PKC) isoforms [62,63]. RT4 cells treated with 10 nM and 100 nM fMLF for 6 h demonstrated similar dynamics of Fpr2, CCR2, CXCR4, and PKCβ levels. These relationships between fMLF-stimulated Fpr2 and CCR2, CXCR4, and PKCβ were confirmed by pretreating RT4 cells with PBP10 (Figure 4a,b,e–g). From these results using an in vitro RT4 schwannoma model, we can assume that chemokine receptors CCR2 and CXCR4 may serve as co-receptors of Fpr2 when Schwann cells of the distal nerve stump are stimulated with formylated peptides released from injured axons.

Ligands activating Fpr2 and chemokine receptors CCR2 and CXCR4 are associated with cytoskeletal changes and chemotaxis of various types of cells including Schwann cells [26,36,43,64,65]. In addition, PKCβ is involved in signaling pathways that regulate actin cytoskeletal reorganization associated with changes in cell shapes [37]. The detected effects of fMLF on Fpr2, CCR2, and CXCR4 levels, along with similar alterations of PKCβ, suggest cooperation between these receptors in the course of cytoplasmic processes in RT4 cells following fMLF stimulation. However, our previously published results revealed no effect of low fMLF concentrations (100 nM and 10 µM) on the number of RT4 cell processes after 1 h and 6 h exposure. Nevertheless, fMLF stimulation at 100 nM for 24 h significantly increased the number of cytoplasmic processes per cell, but at higher concentrations (10 µM and 50 µM) the number of processes was reduced [45]. The differences in the time-course of the fMLF effect can be explained by assuming that molecular regulations precede their morphological manifestation; however, further experiments are needed before we can support that conclusion.

## 5. Conclusions

RT4 schwannoma cells were used as an experimental in vitro model for Schwann cells to study the effect of fMLF on the regulation of Fpr2 and TLR9—receptors for mitochondrial peptides and DNA, respectively. fMLF, a prototypical formylated mitochondrial peptide, upregulated Fpr2 only at low concentrations (10 nM and 100 nM) for 6 h, but upregulation at higher concentrations (10 µM and 50 µM) required inhibition of the cleaved active form of TLR9. The results revealed for the first time that at high concentrations, fMLF can modulate inflammatory profiling of Schwann cells via TLR9. On the other hand, 10 nM and 100 nM fMLF stimulation upregulated Fpr2 in RT4 schwannoma cells, and also increased levels of chemokine receptor CCR2 and CXCR4 as well as PKCβ. This suggested the involvement of Fpr2 activated by a lower concentration of fMLF in the intracellular machinery associated with movement of Schwann cells or development of their cytoplasmic processes that are important for axon regeneration.

## Figures and Tables

**Table 1:** The treatment schedule to study the formylated peptide-mediated stimulation of Fpr2 and TLR9 in RT4 cells induced using various concentrations of fMLF and pretreated with inhibitors.

	fMLF-Treatment	10 nM	100 nM	1 μM	10 μM	50 μM
**Fpr2**	1 h	−	+	−	+	+
	6 h	+	+	+	+	+
	1 μM CQ + 1 h	−	+	−	+	+
	1 μM CQ + 6 h	−	+	−	+	+
	1 μM PBP10 + 6 h	+	+	+	−	−
**TLR9**	1 h	−	+	−	+	+
	6 h	−	+	−	+	+
	1 μM CQ + 1 h	−	+	−	+	+
	1 μM CQ + 6 h	−	+	−	+	+

**Table 2:** List of primary antibodies used for Western blot analysis.

Primary Antibody	Type of Antibody	Dilution	Cat. No/Producer
Fpr2	polyclonal rabbit	1:500	NLS1878/Novus
TLR9	monoclonal mouse	1:1000	NBP2-24729/Novus
CCR2	polyclonal rabbit	1:2000	NBP1-48337/Novus
CXCR4	polyclonal rabbit	1:2000	LS-C417098/LifeSpan
phospho-p38	monoclonal mouse	1:500	4511/Cell Signaling
NFκB	monoclonal rabbit	1:1000	8242/Cell Signaling
phospho-NFκB	monoclonal rabbit	1:1000	3033/Cell Signaling
IL-6	polyclonal rabbit	1:1000	ARC0062/Invitrogen
PKCβ	polyclonal rabbit	1:2000	NBP2-19846/Novus
PKCδ	monoclonal mouse	1:500	Sc-8402/Santa Cruz
